# Necrotizing Granulomatous *Pneumocystis* Infection Presenting as a Solitary Pulmonary Nodule: A Case Report and Review of the Literature

**DOI:** 10.1155/2022/7481636

**Published:** 2022-07-28

**Authors:** Mansur Assaad, Mohamed Swalih, Apurwa Karki

**Affiliations:** Department of Pulmonary and Critical Care Medicine, Guthrie Robert Packer Hospital, Sayre, PA, USA

## Abstract

*Pneumocystis jirovecii* is an opportunistic fungus that is classically associated with pneumonia in immunocompromised patients, particularly those with human immunodeficiency virus and acquired immunodeficiency syndrome (HIV/AIDS). However, this infection is now more commonly seen in those with malignancy, particularly lymphoproliferative disorders. Classic image findings with *Pneumocystis jirovecii* pneumonia (PJP) include bilateral ground-glass opacities with or without cyst formation. Up to 5% of patients with PJP may present with atypical image findings, specifically nodular opacities or masses thought to represent granulomatous inflammation. The differential diagnosis for a new solitary pulmonary nodule in an immunocompromised patient is broad. One must first rule out a recurrence of malignancy or new primary malignancy. In our patient's case, two nondiagnostic bronchoscopies with EBUS-TBNA eventually led to a surgical resection, which revealed a diagnosis of nodular necrotizing granulomatous *Pneumocystis jirovecii*. The diagnostic yield from EBUS is not well established, and most cases require surgical biopsy for definitive diagnosis. Further data regarding the use of EBUS-TBNA in diagnosing granulomatous PJP is needed.

## 1. Introduction

*Pneumocystis jirovecii* is an opportunistic fungus that is classically associated with pneumonia in immunocompromised patients, especially those with human immunodeficiency virus and acquired immunodeficiency syndrome (HIV/AIDS) [1]. However, this infection is now more commonly seen in those with malignancy, particularly lymphoproliferative disorders [1]. Classic imaging findings with *Pneumocystis jirovecii* pneumonia (PJP) include bilateral ground-glass opacities with or without cyst formation [1]. Up to 5% of patients with PJP may present with atypical imaging findings, specifically nodular opacities or masses thought to represent granulomatous inflammation [2]. Herein, we present an interesting case of granulomatous PJP presenting as a progressively enlarging solitary pulmonary nodule.

## 2. Case Report

**Figure 1:**
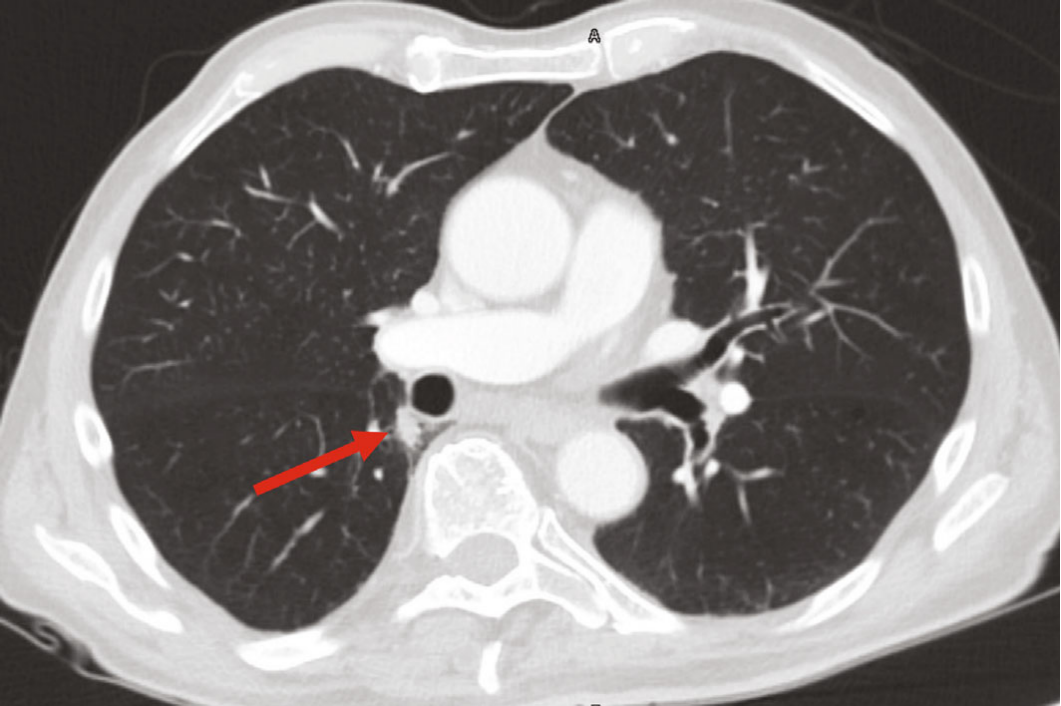
CT of the chest following treatment for peripheral T-cell lymphoma showing a new 1.2 cm nodule in the medial right lower lobe just posterolateral to the distal bronchus intermedius. The nodule appears solid with smooth and spiculated margins and surrounding subtle interstitial thickening. No other obvious abnormalities were noted on CT imaging.

**Figure 2:**
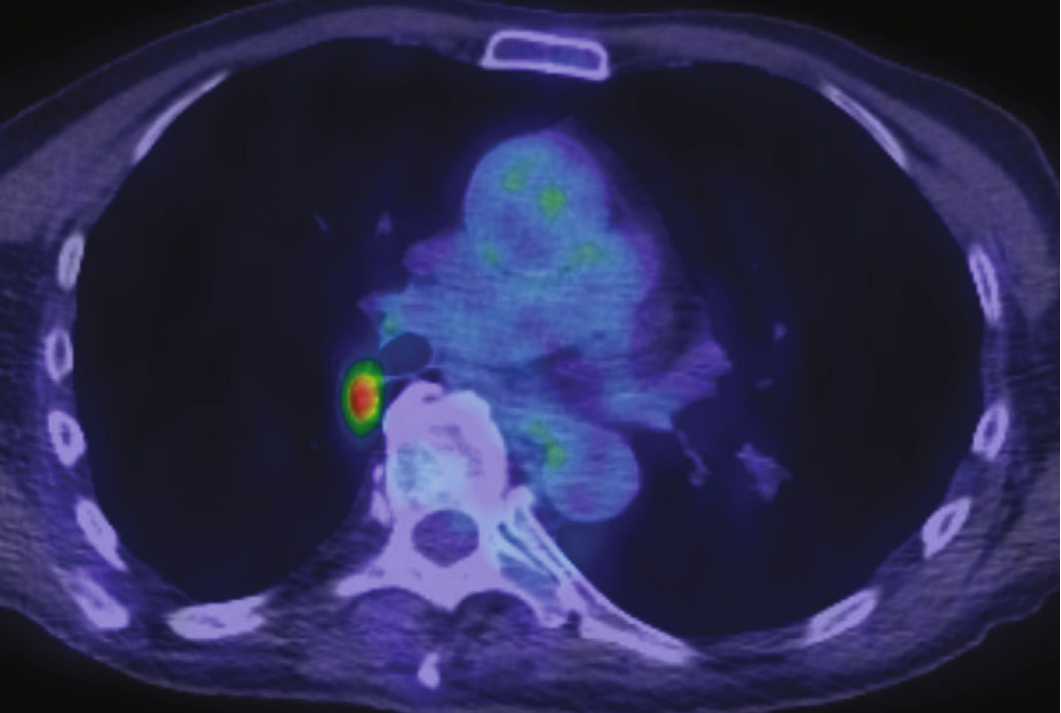
PET-CT scan showing the hypermetabolic nodule with a SUV of 5.7, concerning for a possible primary pulmonary malignancy.

A 76-year-old man with a history of peripheral T-cell lymphoma treated with six cycles of cyclophosphamide, doxorubicin, vincristine, and prednisone (CHOP) was referred for abnormal imaging. Initial imaging showed enlarged mediastinal lymphadenopathy, particularly within the right paratracheal and subcarinal regions, as well as prominent mesenteric, retroperitoneal, and pelvic lymphadenopathy. A surveillance computed tomography (CT) of the chest, abdomen, and pelvis performed two months after therapy showed dramatic improvement. However, a new 1.2 cm pulmonary nodule was seen in the superior-medial aspect of the right lower lobe just lateral to the distal bronchus intermedius (Figure 1). A subsequent positron emission tomography-CT (PET-CT) scan revealed this nodule to be hypermetabolic with a standardized uptake value (SUV) of 5.7, raising the suspicion for a possible primary pulmonary malignancy (Figure 2).

**Figure 3:**
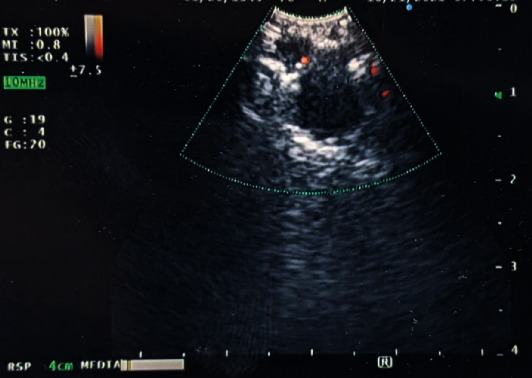
Bronchoscopy with EBUS showing an enlarged right hilar nodule.

The patient underwent bronchoscopy with linear endobronchial ultrasound (EBUS). After passing the scope into the distal bronchus intermedius and rotating the probe to face the posterior wall, the pulmonary nodule was identified on ultrasound. A 22-guage needle was used to obtain tissue. Cytology showed no evidence of malignant cells, and flow cytometry showed no discrete clonal B-cell or atypical T-cell population. No tissue was sent for culture. The patient underwent follow-up CT imaging two months later, which showed an enlargement of the nodule to 1.8 cm and two new 1 cm nodules in the peripheral basal right lower lobe. A repeat EBUS was performed, and the area of interest was identified (Figure 3). A 22-guage needle was used to obtain tissue from the same site; this time, suction was used, and up to fifteen passes with the needle were taken with each aspiration. Rapid on-site evaluation (ROSE) did not confirm the presence of lymphocytes. Cytology was again nondiagnostic. No tissue was sent for culture.

**Figure 4:**
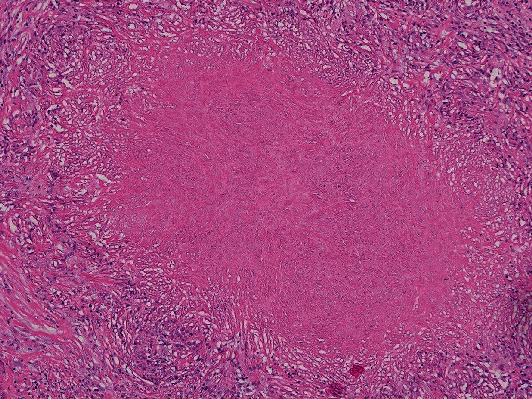
Hematoxylin and eosin stain of surgical biopsy showing multiple necrotizing granulomas, some of which are surrounded by organizing pneumonia.

**Figure 5:**
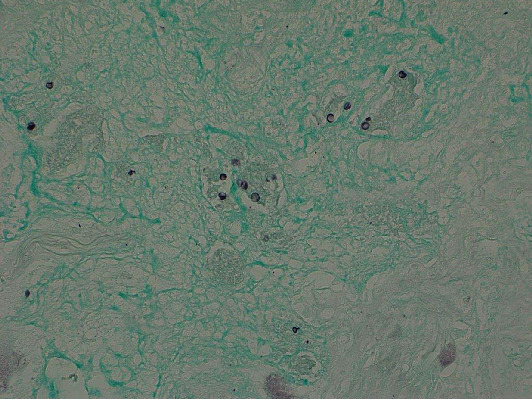
Gomori methenamine silver stain showing organisms compatible with *Pneumocystis jirovecii*.

The patient was referred to thoracic surgery. He underwent a right video-assisted thoracoscopic surgery (VATS) with successful resection of the dominant right hilar nodule and two smaller basilar nodules. Pathology from the dominant nodule revealed multiple necrotizing granulomas, some of which were surrounded by organizing pneumonia (Figure 4). Acid-fast bacilli stain was negative. Grocott methenamine silver (GMS) stain demonstrated clusters of organisms compatible with *Pneumocystis* in the center of these granulomas (Figure 5). On follow-up, the patient remained asymptomatic. He declined treatment with trimethoprim-sulfamethoxazole (TMP-SMX) and elected to pursue serial CT scans to monitor for recurrence. A repeat CT chest 6 months later showed no evidence of recurrence.

## 3. Discussion

PJP classically presents with an image pattern of bilateral alveolar and interstitial infiltrates with a perihilar distribution and upper-lobe predominance [3, 4]. However, atypical radiographic findings, specifically an enlarging solitary pulmonary nodule, have been reported in a handful of cases. Furthermore, granulomatous inflammation is a rare pathologic finding with PJP and comprises 3-5% of the cases [3, 5]. Several host factors may play a role in the pathogenesis of granulomatous PJP infection, including active malignancy, long-term corticosteroid use, and the presence of immune reconstitution inflammatory syndrome, and, as in this patient, prior treatment of lymphoma with CHOP therapy [3].

Diagnosis can be challenging. Compared with classic PJP with alveolar infiltrates, organisms are rarely found in the alveolar lumen with granulomatous PJP, making the diagnostic yield from bronchoalveolar lavage (BAL) in these patients very poor [5, 6]. Furthermore, the presence of an enlarging solitary pulmonary nodule in a patient previously treated for a lymphoproliferative disorder likely requires more tissue acquisition to assess for recurrence of disease, development of a new primary malignancy, or, as in this case, development of an opportunistic infection. Bronchial brushings, needle aspiration, and transbronchial and image-guided biopsy are considered unfavorable for diagnosis [7]. Routine fungal cultures from EBUS-transbronchial needle aspirations (EBUS-TBNA) are generally considered unhelpful given low yield and high rate of contamination. However, there have been rare case reports of PJP diagnosed by EBUS-TBNA of mediastinal lymphadenopathy. Moualla and Saeed reported an immunosuppressed 37-year-old patient due to a prior kidney transplant who developed dyspnea and fever [8]. Imaging showed bilateral pulmonary nodules and mediastinal adenopathy, and EBUS-TBNA of a mediastinal lymph node later confirmed granulomatous PJP. Further data regarding diagnostic yield of EBUS-TBNA in granulomatous PJP is needed. Given the overall poor yield from bronchoscopic sampling, surgical lung biopsy is cited as the most accurate technique for diagnosing granulomatous PJP presenting as a solitary pulmonary nodule [9, 10]. Although no formal guidelines regarding the management of granulomatous PJP exist, therapy with TMP-SMX is generally offered [7]. As with our patient, surgical resection can be both diagnostic and potentially therapeutic. Serial imaging to monitor for recurrence of disease is suggested. Our patient had a repeat CT chest 6 months after surgery, which showed no evidence of recurrence, and he remained asymptomatic at that time.

Literature review yielded eight peer-reviewed published cases of nodular granulomatous PJP between the years of 2014 and 2021. Clinical findings including demographics, symptoms, primary diagnosis, radiology, diagnostic procedure, and antibiotic treatment can been seen in Table 1. Patients were predominantly male. Age ranged from 37 to 69 years. Most patients (75%) presented with respiratory symptoms, including dyspnea or cough, while the remaining patients were asymptomatic. The primary diagnosis varied significantly and included peripheral T-cell lymphoma, diffuse large B-cell lymphoma, rectal cancer, chronic lymphocytic leukemia, HIV, and postkidney transplant. One patient had no significant history other than active tobacco abuse. CT images for all patients showed either a solitary pulmonary nodule or multiple bilateral nodules with one patient displaying enlarged mediastinal lymphadenopathy. Most patients (75%) were diagnosed via VATS with surgical resection; however, one patient was diagnosed with GMS stain from BAL and another patient was diagnosed via EBUS-TBNA of an enlarged mediastinal lymph node. Most patients (63%) responded well to TMP-SMX (in addition to surgical resection for those who received it). One patient responded well to atovaquone, and another patient responded well to pentamidine. One patient was managed successfully with surgical resection alone.

## 4. Conclusion

The differential diagnosis for a new solitary pulmonary nodule in an immunocompromised patient is broad. One must first rule out a recurrence of malignancy or new primary malignancy. The clinician must also be aware of atypical presentations of opportunistic infections. In our case, two nondiagnostic bronchoscopies with EBUS-TBNA eventually led to a surgical resection, which revealed a diagnosis of nodular necrotizing granulomatous *Pneumocystis jirovecii*. The diagnostic yield from EBUS is not well established, and most cases require surgical biopsy for definitive diagnosis. Further data regarding the use of EBUS-TBNA in diagnosing granulomatous PJP is needed.

## Figures and Tables

**Table 1:** Clinical characteristics and diagnosis of eight patients with nodular granulomatous *Pneumocystis jirovecci* pneumonia.

Patient number	Dako et al. 2019	Lam et al. 2013	Patel et al. 2016	Kim et al. 2016	Kim et al. 2015	Paul 2018	Moualla and Saeed 2014	Dai et al. 2021
Age	49	49	61	47	69	68	37	59
Gender	Female	Female	Male	Male	Female	Male	Male	Male
Symptoms	Dyspnea, cough	Cough, weight loss	Dyspnea, cough	Cough	Asymptomatic	Asymptomatic	Dyspnea, fever	Cough
Primary diagnosis	Peripheral T-cell lymphoma	Active smoker	DLBCL	HIV	DLBCL	Rectal cancer	Kidney transplant	CLL
Radiology	Multiple nodules	SPN	SPN	Multiple nodules	SPN	SPN	Multiple nodules, mediastinal LAD	SPN
Diagnostic procedure	BAL	VATS	VATS	VATS	VATS	VATS	EBUS-TBNA	VATS
Antibiotic treatment	TMP-SMX	TMP-SMX	Atovaquone	TMP-SMX	TMP-SMX	None	TMP-SMX	Pentamidine

Data pulled from references 1–3, 5–9. BAL: bronchoalveolar lavage; SPN: solitary pulmonary nodule; DLBCL: diffuse large B-cell lymphoma; VATS: video-assisted thoracoscopic surgery; HIV: human immunodeficiency virus; LAD: lymphadenopathy; EBUS-TBNA: endobronchial ultrasound-transbronchial needle aspiration; CLL: chronic lymphocytic leukemia; TMP-SMX: trimethoprim-sulfamethoxazole.
